# Clinical outcomes of the tunnelized-facial artery myomucosal island flaps in the oral cavity and comparison with cutaneous flaps in an animal model

**DOI:** 10.3389/fonc.2026.1760967

**Published:** 2026-04-07

**Authors:** Zengyu Chen, Yiyao Zhu, Min Huang, Yijun Deng, Le Yang, Guiqing Liao, Sien Zhang

**Affiliations:** 1 Hospital of Stomatology, Sun Yat-sen University, Guangzhou, Guangdong, China; 2 Guangdong Provincial Key Laboratory of Stomatology, Guangzhou, Guangdong, China; 3 Guanghua School of Stomatology, Sun Yat-sen University, Guangzhou, Guangdong, China

**Keywords:** oral squamous cell carcinoma, plastic surgery procedures, reconstructive surgery, surgical flaps, tongue neoplasms

## Abstract

**Objective:**

This study aims to evaluate the clinical efficacy of tunnelized-facial artery myomucosal island flaps (t-FAMMIF) in reconstructing mucosal defects following oral cancer surgery and compare the characteristics between mucosal and cutaneous flaps.

**Methods:**

A total of 23 patients who underwent t-FAMMIF reconstruction were enrolled and monitored for functional recovery and complication resolution. Using propensity score matching, 20 cases were paired with a cohort of patients receiving cutaneous flaps (n=20 per group). Sensory function was assessed using the Semmes-Weinstein monofilament test and two-point discrimination tests, while motor function was evaluated with the Water Swallowing Test. Facial nerve function was assessed by House-Brackmann grading scale, wireless surface electromyography, and FaceGram software. Mouth opening was measured by maximum inter-incisal opening. Additionally, to explore the mechanisms underlying differential functional recovery, mucosal and cutaneous flaps were compared in a beagle dog model, with subsequent histological analysis.

**Results:**

Compared to the cutaneous flap group, the t-FAMMIF group exhibited superior aesthetic and sensory outcomes, with facial nerve dysfunction and trismus largely resolved by 6 months. Histological analysis in the animal model revealed that mucosal flaps preserved minor salivary glands and exhibited significantly reduced scar formation, providing a tissue-level explanation for the enhanced sensory recovery observed clinically.

**Conclusion:**

T-FAMMIF is an effective option for reconstructing small to moderate mucosal defects. Compared to cutaneous flaps, it provides advantages including superior aesthetic results, enhanced sensory recovery, minimized scarring, and preservation of salivary gland function.

## 1 Introduction

Oral cancer is one of the most common head and neck malignancies. According to the Global Cancer Observatory, in 2022, there were 389,846 incident cases of oral cancer worldwide, with the majority (66.3%) occurring in Asia (1). In China, the incidence of oral cancer has steadily increased, reaching 4.61 per 100,000 persons in 2022 (2). These epidemiological figures underscore the significant burden of oral cancer as well as the need for prompt, effective post-resection reconstruction in a large patient population. Owing to the tumor’s anatomical location, the post-tumor-resection defect presents a complex clinical challenge as it involves not only aesthetic appearance but also vital functions, such as sensation, speech, and swallowing. Consequently, the long-term goal of reconstructive surgery is to select an appropriate repair method and restore the preoperative morphology and function to the greatest extent possible.

Despite being the most common method for repairing mucosal defects in reconstructive procedures following oral cancer surgery, cutaneous flaps have notable disadvantages, including the risks of chronic inflammation and secondary tumor formation (3–6), scar hypertrophy that may impair movement function (7), discomfort caused by hair growth, and difficulty in shaping, particularly for small or delicate defects, such as those on the tongue tip (8–10).

In 1992, Pribaz et al. introduced the facial artery musculomucosal (FAMM) flap. However, its vascular pedicle was dissected only as far as the floor of the vestibular sulcus, which limited the radius of rotation (11). More recently, the FAMM flap has undergone continuous improvements and is currently suitable for various maxillofacial reconstruction scenarios (12, 13). Among these, one technique involves the dissection of the facial artery up to its origin from the external carotid artery in the neck and bypassing the mandible through a floor-of-mouth tunnel. Thus, the tunnelized-facial artery myomucosal island flap (t-FAMMIF) offers a larger radius of rotation to correct a broader range of defects. This technique is particularly effective for postoperative defects following resection of T2–T3 oral cancer.

Herein, we describe the surgical procedure of t-FAMMIF and assess its outcomes in clinical cohort. Importantly, while previous studies have focused on clinical outcomes, the biological mechanisms underlying the functional advantages of mucosal flaps remain largely unexplored. To address this gap, we developed a beagle dog model to directly compare mucosal and cutaneous flaps, with subsequent histological analysis aimed at elucidating the tissue-level basis for their differences. This combined clinical and histological approach provides novel insights into optimizing post-resection reconstruction strategies.

## 2 Materials and methods

### 2.1 Patient selection and follow-up

The t-FAMMIF cohort was selected from consecutive patients who were treated at the Hospital of Stomatology, Sun Yat-sen University (Guangdong, People’s Republic of China) between February 2020 and March 2023. The inclusion criteria were as follows: (a) preoperative diagnosis of oral squamous cell carcinoma; (b) the mucosal defect following tumor resection involved the tongue, floor of mouth, buccal mucosa, or lip; (c) no evidence of cervical lymph node or distant metastasis on preoperative examination; and (d) application of an ipsilateral t-FAMMIF to reconstruct the mucosal defect. The exclusion criteria included (a) through-and-through buccal defects (i.e., combined mucosal-cutaneous defects); (b) bone defects; (c) death from causes unrelated to tumor progression; and (d) interrupted follow-up. No specific age cutoff was used for patient selection. The detailed surgical procedure was described in a subsection.

To evaluate the clinical efficacy of t-FAMMIF, a commonly used cutaneous flap (anterolateral thigh flap, ALT) was employed as the control group based on the same criteria described above. Propensity score matching (PSM) was applied to create a balanced cutaneous flap group based on baseline characteristics, including age, sex, pTNM stage, and neck dissection status. A matched cutaneous flap group consisting of 20 patients was obtained using a 0.05 caliper and a 1:1 matching ratio.

Patients underwent standardized testing and follow-up assessments prior to surgery and at 1, 3, 6, and 12 months postoperatively. Progression-free survival (PFS) and functional recovery assessments (sensation and swallowing) were performed in both groups to compare efficacy, while complications assessments (facial nerve function and mouth opening) were performed in the t-FAMMIF group to assess procedure-specific morbidity.

This study was approved by the ethics committee of Guanghua School of Stomatology, Sun Yat-sen University, Guangdong, People’s Republic of China. All patients provided written informed consent for participation in the study, and the study was conducted in strict adherence to the tenets underlying the Declaration of Helsinki.

### 2.2 Surgical procedure of the t-FAMMIF

**Figure 1:**
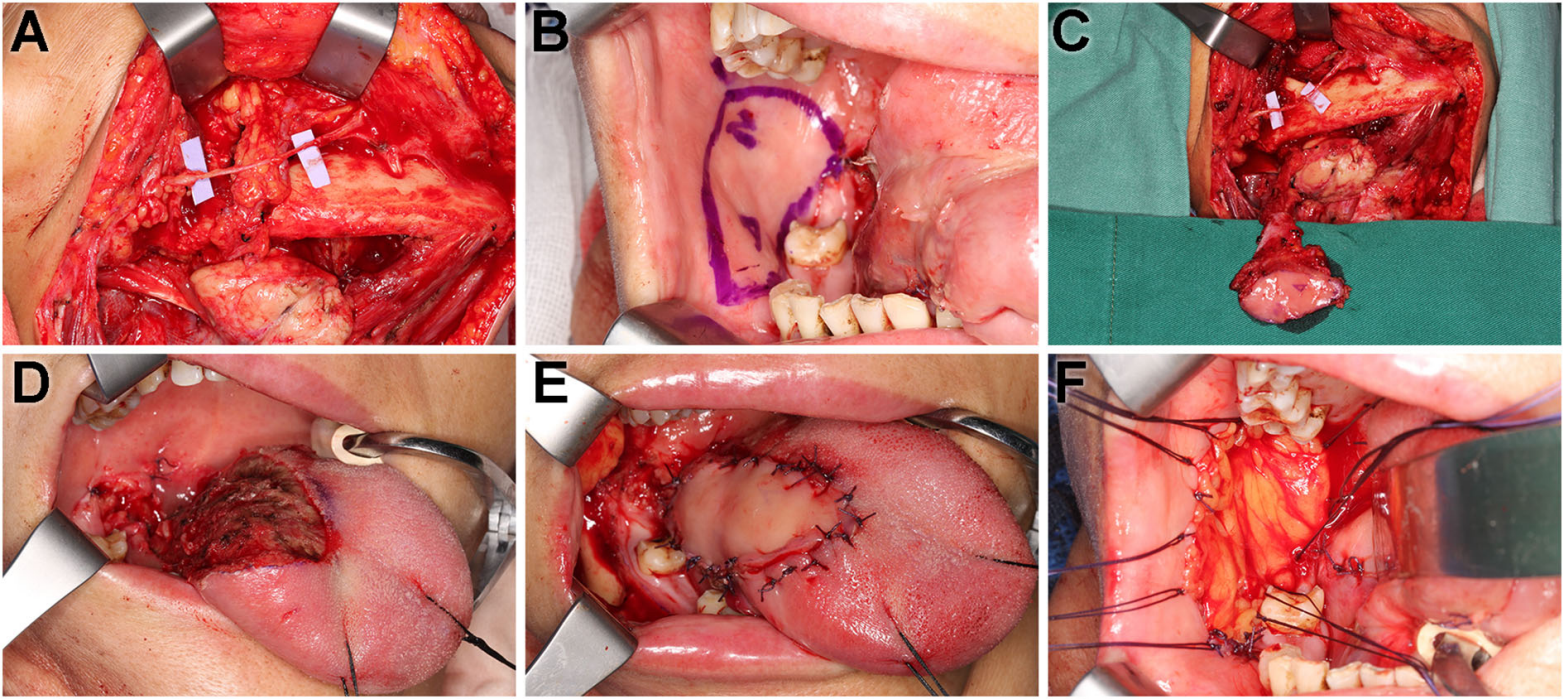
Surgical procedure of t-FAMMIF. **(A)** The vascular pedicle and the marginal mandibular branch of the facial nerve were dissected. **(B)** Intraoral design of the t-FAMMIF: Markers for indicating the surface projection of the facial artery, vein, and the parotid duct orifice. **(C)** Harvest of the t-FAMMIF. **(D)** The defect after tumor resection. **(E)** Tongue reconstruction with t-FAMMIF. **(F)** The donor site was repaired with the buccal fat pad.

An incision was made 2 cm below the lower edge of the mandible, and the skin, subcutaneous fat, and platysmal muscle layer were sequentially incised. The platysmal flap was elevated to expose the facial artery and vein along the anterior border of the masseter muscle, near the lower edge of mandible. The marginal mandibular branch of the facial nerve should be identified and exposed prior to vessel dissection, ensuring its visualization throughout the procedure to prevent iatrogenic injury. The blood vessels entering and exiting the submandibular gland were transected whereas the facial artery, facial vein, and the marginal mandibular branch of the facial nerve were preserved. The dissection was extended along the distal facial artery and vein toward the buccal branch of the vessels (**Figure 1A**).

The flap contour was designed on the buccal mucosa along the facial artery. The upper edge was positioned at least 5 mm away from the orifice of the parotid duct, and the anterior edge was placed at least 10 mm from the oral commissure. The remaining borders could be extended posteriorly to the pterygomandibular ligament and inferiorly to the mandibular vestibule and thereby customized to match the size of the defect (**Figure 1B**).

The buccal mucosa was incised along the intraoral incision design, and the submucosal buccinator muscle was dissected until the vascular pedicle was identified. Then, the distal ends of the artery and vein were ligated, and the vascular pedicle was carefully dissected to its origin from the external carotid artery and retromandibular vein, ensuring that it remained on the flap side with sufficient tissue surrounding the pedicle. Following this, the preparation of the t-FAMMIF was considered complete (**Figure 1C**).

A tunnel was created through the floor of the mouth, and the prepared t-FAMMIF was transferred to the intraoral defect, trimmed, and sutured in place. The donor site was packed with the buccal fat pad and iodoform gauze (**Figures 1D–F**).

### 2.3 Functional assessment

#### 2.3.1 Sensory function

All tests were conducted on the central surface of the flaps in a quiet room by a single trained examiner who was blinded to the patient’s group assignment. Participants were instructed to close their eyes. Tactile sensation was assessed using the Semmes-Weinstein monofilament test (Premier Products, USA), and two-point discrimination was evaluated using vernier calipers. The stimulation thresholds were recorded. Recovery was defined as the presence of any detectable sensation using the maximum stimulus (300 g for tactile sensation and 15 mm for two-point discrimination).

#### 2.3.2 Swallowing function

Patients were required to continuously drink 30 mL water as quickly as possible, and the swallowing function was graded based on the swallowing efficacy and the occurrence of coughing or choking as follows: Grade I (swallow smoothly in a single attempt), Grade II (swallow in more than one attempt without choking), Grade III (swallow in a single attempt with choking), Grade IV (swallow in more than one attempt with choking), and Grade V (frequently chokes and is unable to swallow all the water).

#### 2.3.3 Facial nerve function

The facial nerve function was evaluated by three methods.

The House–Brackmann grading system (HBGs) was used for subjective assessment of facial nerve function (14). A minimum of three experienced clinicians independently evaluated each patient’s facial movements, including resting state, eyelid closure, cheek puffing, smiling with teeth exposed, and lip pursing.

Facial nerve function, particularly the buccal and marginal mandibular branches, was assessed using a wireless surface electromyography (sEMG) system (Trigno™ Wireless Systems, Delsys Inc., USA). The normalization procedures were performed consistently with previously described protocols (15). The root mean square (RMS) values for the operated sides during lip pursing were recorded and analyzed.

Images of the smiling expression of the patients were captured under standardized conditions, and, using FaceGram software (v1.0, Massachusetts Eye and Ear Infirmary, Boston, MA, USA), the angle between the angulus oris and the midline was measured on both the operated and healthy sides.

#### 2.3.4 Mouth opening

The maximum inter-incisal opening (MIO) was measured using a vernier caliper to evaluate the patient’s mouth-opening. Patients in the t-FAMMIF group were instructed to begin intermittent mouth-opening exercises 2 weeks after surgery, for a total of 30–60 minutes per day, until the MIO returned to near normal levels (> 35 mm).

### 2.4 Animal model construction

All animal experiments were approved by the Ethics Committee of Laboratory Animal Center, Sun Yat-sen University, Guangdong, People’s Republic of China. Six beagle dogs were randomly assigned to either the t-FAMMIF group (n = 3) or the submental skin-island flap (SMIF) group (n = 3). Anesthesia was induced with xylazine hydrochloride and maintained via intraperitoneal administration of 3% sodium pentobarbital. Perioperative penicillin was administered. In both groups, a standardized 5 cm × 3 cm mucosal defect was created on the lateral border of the tongue, and flaps with identical size were harvested based on their respective vascular pedicles.

Postoperatively, animals wore a protective collar for 2 weeks, and analgesia was provided via nasogastric administration of ibuprofen. For histological analysis, tissue specimens were collected from the flap edges intraoperatively (as preoperative baseline) and at 3, 6, and 12 months postoperatively.

### 2.5 Histological assessment

All the specimens underwent a multi-step dehydration process with ethanol, followed by paraffin embedding and sectioning. Standard hematoxylin–eosin staining was performed for histological observation and assessment. Using a mouse anti-aquaporin-5 antibody (Santa Cruz Biotechnology, 1:200), aquaporin-5 expression in the minor salivary glands was evaluated by immunohistochemistry (IHC) to assess the secretory function of the glands. Furthermore, Masson’s trichrome staining was performed to detect collagen accumulation in the flap and to evaluate scarring and tissue tenderness. Five random fields per slide were captured at 20× magnification. The proportion of blue-stained collagen area was measured using ImageJ software (v1.8, NIH, Bethesda, USA).

### 2.6 Statistical analysis

All statistical analyses were performed using IBM SPSS Statistics (Version 25.0, IBM, USA). Baseline characteristics of the matched groups were compared using the χ2 test or t-test. The log-rank test and Kaplan-Meier plot were employed to compare survival outcomes between the two groups. Functional data from the t-FAMMIF and cutaneous flap groups were analyzed with the Mann-Whitney U test. Complications in the t-FAMMIF cohort were analyzed using the Friedman test or one-way repeated measures ANOVA. Statistical significance was set at *p* ≤ 0.05 (two-tailed), with corresponding 95% confidence intervals.

## 3 Results

### 3.1 Clinical application and efficacy evaluation of the ipsilateral t-FAMMIF in reconstructing mucosal defects following surgery for oral cancer

#### 3.1.1 Participants’ characteristics

A total of 23 patients were included in the t-FAMMIF cohort, consisting of 14 men and 9 women, with a mean age of 52.35 years. Patient characteristics are presented in **Table 1**.

**Table 1:** Patient characteristics of the t-FAMMIF cohort (n=23).

Patient no.	Age (y)	Gender	Site	cTNM	pTNM	Neck dissection
1	55	Female	Tongue	T2N0M0	T2N0M0	Yes
2	52	Male	Tongue	T2N0M0	T2N0M0	Yes
3	55	Male	Tongue	T3N0M0	T3N0M0	Yes
4	64	Female	Tongue	T2N0M0	T2N0M0	Yes
5	48	Male	Lip	T2N0M0	T2N0M0	Yes
6	60	Male	Floor of Mouth	T2N0M0	T2N0M0	Yes
7	56	Male	Floor of Mouth	T2N0M0	T2N0M0	Yes
8	63	Male	Tongue	T2N0M0	T2N0M0	Yes
9	66	Female	Tongue	T2N0M0	T2N1M0	Yes
10	54	Male	Tongue	T2N0M0	T2N0M0	Yes
11	35	Male	Tongue	T2N0M0	T2N0M0	Yes
12	37	Male	Cheek	T2N0M0	T3N2M0	Yes
13	49	Male	Tongue	T2N0M0	T2N0M0	Yes
14	36	Male	Tongue	T2N0M0	T2N0M0	Yes
15	71	Female	Tongue	T2N0M0	T2N0M0	Yes
16	40	Female	Tongue	T2N0M0	T2N1M0	Yes
17	45	Female	Tongue	T1N0M0	T1N0M0	Yes
18	42	Female	Tongue	T2N0M0	T2N0M0	Yes
19	44	Male	Tongue	T2N0M0	T2N0M0	Yes
20	74	Male	Lip	T2N0M0	T2N0M0	No
21	67	Male	Tongue	T2N0M0	T1N0M0	Yes
22	44	Female	Tongue	T1N0M0	T2N0M0	Yes
23	47	Female	Tongue	T1N0M0	T1N0M0	Yes

A propensity score matching method was employed to create a matched cohort for t-FAMMIF and cutaneous flap. Three t-FAMMIF cases failed to be matched, resulting in two paired groups, each with a sample size of 20. Baseline characteristics were comparable between the two groups (**Table 2**).

**Table 2:** The baseline information of the t-FAMMIF and matched cutaneous flap group.

Characteristics	t-FAMMIF (n=20)	Cutaneous flap (n=20)	*p* value
Age (y)	52.30 (11.60)	51.85 (9.03)	0.182
Gender			0.744
Male	13 (65)	12 (60)	
Female	7 (35)	8 (40)	
Smoking			0.527
Yes	9 (45)	11 (55)	
No	11 (55)	9 (45)	
Alcohol			0.723
Yes	6 (30)	5 (25)	
No	14 (70)	15 (75)	
Diabetes			0.231
Yes	0 (0)	3 (15)	
No	20 (100)	17 (85)	
Site			0.170
Tongue	15 (75)	12 (60)	
Lip	3 (15)	1 (5)	
Floor of mouth	2 (10)	3 (15)	
Cheek	0 (0)	4 (20)	
pT stage			1.000
T1	1 (5)	2 (10)	
T2	17 (85)	16 (80)	
T3	2 (10)	2 (10)	
pN stage			1.000
N0	17 (85)	17 (85)	
N1	2 (10)	2 (10)	
N2	1 (5)	0 (0)	
N3	0 (0)	1 (5)	
pM stage			NA
M0	20 (100)	20 (100)	
Neck dissection			1.000
Yes	19 (95)	19 (95)	
No	1 (5)	1 (5)	
Radiotherapy			1.000
Yes	4 (20)	4 (20)	
No	16 (80)	16 (80)	
Chemotherapy			1.000
Yes	1 (5)	1 (5)	
No	19 (95)	19 (95)	

Continuous features are shown by mean with standard deviation in brackets. Categorical features are shown by frequency and corresponding proportion in each group. NA: Not available.

#### 3.1.2 Functional assessment

**Figure 2:**
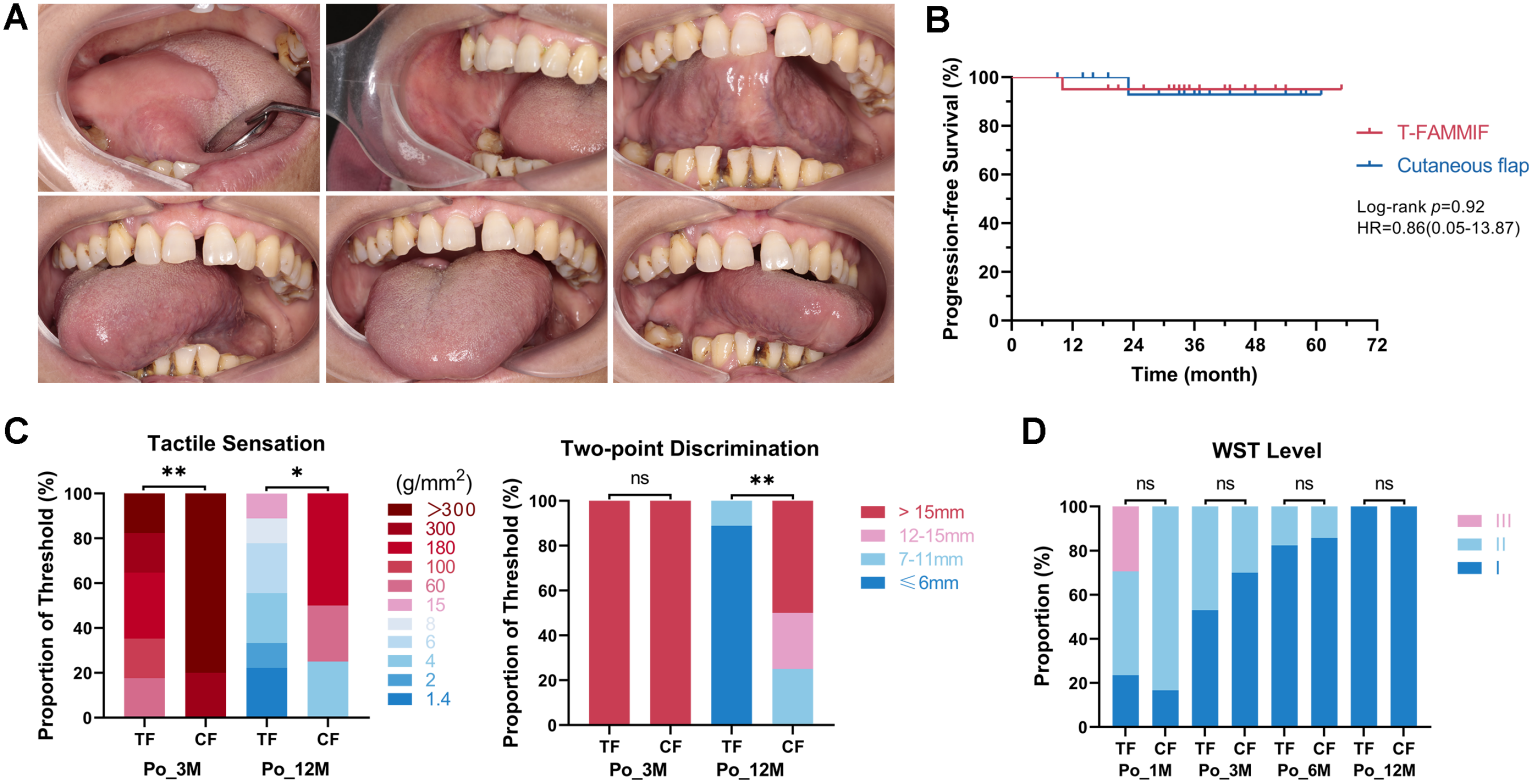
Aesthetic and functional assessment. **(A)** The mucosal flap was red in color and soft in texture with good movement function and the well recovered donor site. **(B)** The Kaplan-Meier curve of the PFS of the t-FAMMIF and cutaneous flap group. **(C)** The results of tactile sensation and two-point discrimination assessment. **(D)** The Water Swallowing Test results. TF, t-FAMMIF; CF, cutaneous flap; Pre-, preoperatively. Po-1M to Po-12M, 1 to 12 months post-operatively. WST, the water swallowing test. (**, *p* ≤ 0.01. *, *p* ≤ 0.05. ns, *p* > 0.05).

Functional assessments were conducted on patients in both the t-FAMMIF and cutaneous flap groups. Six months after surgery, the reconstructed tongues exhibited satisfactory appearance and good mobility. No deformity was detected at the donor sites, and no food residue was observed in the vestibular sulcus. No complications such as bleeding or wound dehiscence occurred in the recipient area. (**Figure 2A**). The median follow-up time was 34.5 months (range, 10–65 m) in the t-FAMMIF group and 35 months (range, 9–61 m) in the matched cutaneous flap group. Nine patients in the t-FAMMIF group and ten patients in the matched cutaneous flap group completed at least 36 months of follow-up. During the follow-up period, one patient in cutaneous flap group developed contralateral cervical lymph node metastasis 23 months after surgery, and one patient in t-FAMMIF group developed ipsilateral cervical lymph node metastasis 10 months after surgery. The 3-year progression-free survival rate was 95.0% in each group, with no significant difference between groups (*p* = 0.920) (**Figure 2B**).

Regarding sensory function, at 3 months postoperatively, the tactile sensation recovery rate was significantly higher in the t-FAMMIF group (80%) than that in the cutaneous flap group (20%) (*p* = 0.010). However, two-point discrimination recovery was not achieved in either group at this time point (>15mm in all patients, *p* = 1.000). At 12 months post-surgery, all patients achieved recovery of tactile sensation (recovery rate: 100% for both groups). Nevertheless, the tactile threshold was significantly lower in the t-FAMMIF group (*p* = 0.040). For two-point discrimination, the recovery rate was 100% in the t-FAMMIF group but only 50% in the cutaneous flap group, and the threshold was also significantly lower in the t-FAMMIF group (*p* = 0.002). Furthermore, in the t-FAMMIF group, sensory thresholds at 12 months were not significantly different from preoperative baseline values (**Supplementary Figure 1**, **Supplementary Table 1**), indicating complete sensory recovery to near-normal levels. These findings indicate that sensory recovery in the t-FAMMIF group was faster and more sensitive compared to the cutaneous flap group (**Figure 2C**, **Table 3**).

**Table 3:** Results of sensory function assessment.

Time	Sensory function assessment	t-FAMMIF (n=20)	Cutaneous flap (n=20)	*p* value
Po-3M	Tactile Sensation (g/mm^2^)	240.00 (189.34)	540.00 (134.16)	0.010**
Po-3M	Two-point Discrimination (mm)	15.00 (0.00)	15.00 (0.00)	1.000
Po-12M	Tactile Sensation (g/mm^2^)	5.31(4.29)	106.00 (88.45)	0.040*
Po-12M	Two-point Discrimination (mm)	4.78 (2.05)	13.5 (2.38)	0.002**

Continuous features are shown by mean with standard deviation in brackets. Po-3M to Po-12M, 3 to 12 months post-operatively. (**, *p* ≤ 0.01. *, *p* ≤ 0.05.).

The Water Swallowing Test (WST) showed that approximately one-third of t-FAMMIF patients experienced choking (grade III) during the first postoperative month. No choking incidents were reported at 3 months postoperatively, and, by 12 months, the water swallowing function of all patients had fully recovered to Grade I. There was no statistically significant difference in swallowing recovery between the t-FAMMIF and cutaneous flap group (**Figure 2D**).

#### 3.1.3 Complications

Complications were assessed in the t-FAMMIF cohort (n = 23). The most common complications associated with t-FAMMIF reconstruction of oral cancer defects were facial nerve dysfunction and trismus.

**Figure 3:**
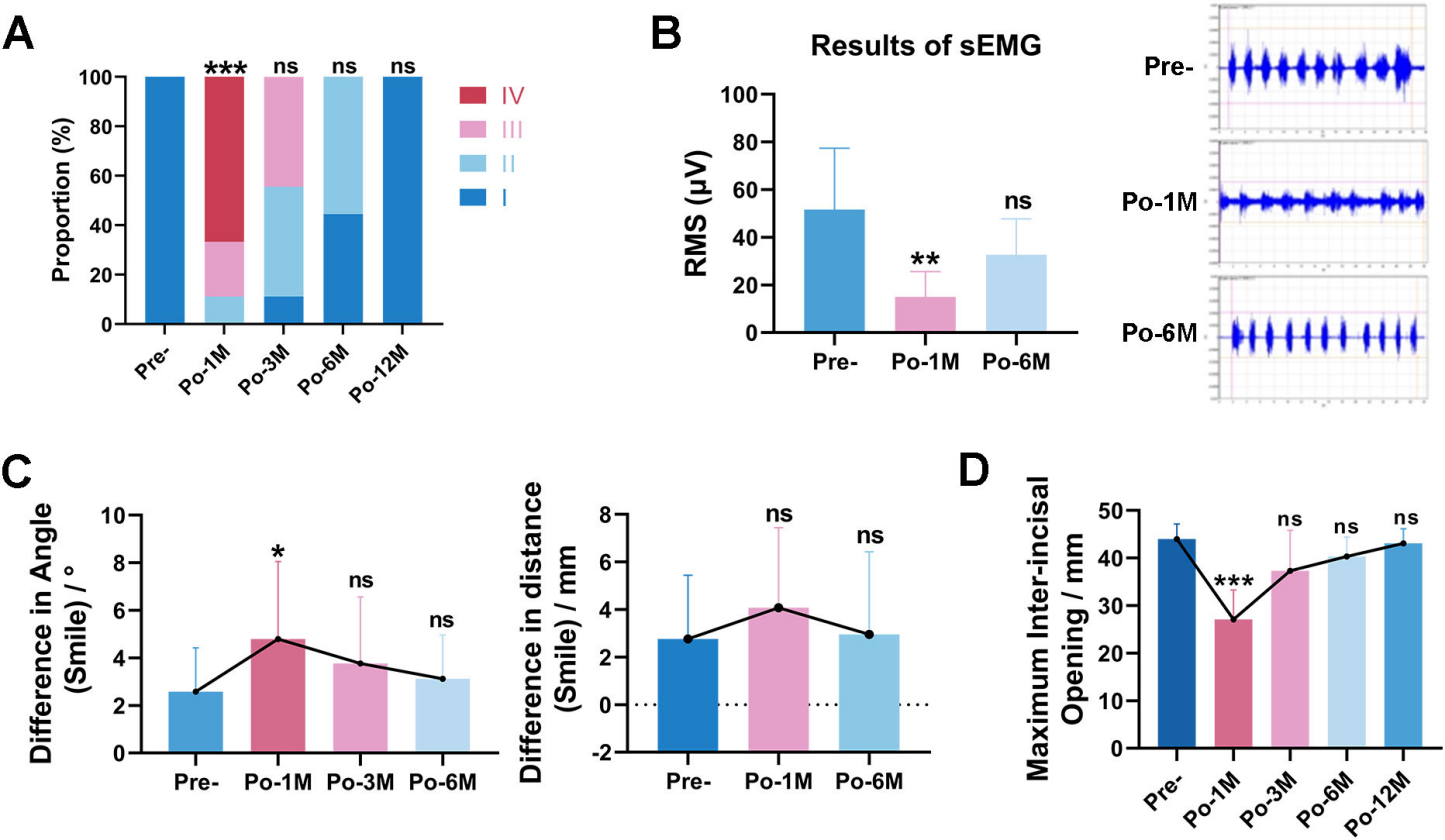
Postoperative complications and recovery status. **(A)** Subjective assessment (HBGs) results of facial nerve function. **(B)** Left: Comparison of root mean square (RMS) results of depressor labii inferior (pursed lips) on the operated side. Right: The sEMG of the operated side of depressor labii inferior in Pre- and Po-1M, Po-6M. **(C)** The angle and distance difference between the operated side and the healthy side. **(D)** Results of mouth opening. HBGs, the House–Brackmann grading system; Pre-, preoperatively. Po-1M to Po-12M = 1 to 12 months post-operatively. sEMG, surface electromyography system. RMS, the root mean square. (***, *p* ≤ 0.001. **, *p* ≤ 0.01. *, *p* ≤ 0.05. ns, *p* > 0.05). All assessments shown are from t-FAMMIF patients only.

According to the House–Brackmann grading system, all patients exhibited normal facial nerve function (Grade I) preoperatively, whereas 1 month after surgery, most patients presented with Grade III/IV facial nerve function, characterized by lip muscle weakness and/or static disfiguring asymmetry (p = 0.000). At the 3 (*p* = 0.113) and 6 month (*p* = 1.000) postoperative evaluations, facial nerve function demonstrated progressive improvement. By 12 months postoperatively, all patients had largely recovered to Grade I facial nerve function (*p* = 1.000) (**Figure 3A**).

The dynamic symmetry of the angulus oris was assessed using sEMG and the FaceGram software. The sEMG data revealed that the RMS amplitude of the labii quadratus lipi (pout) on the operated side was significantly reduced at 1 month postoperatively (*p* = 0.005), but generally returned to baseline level by 6 months (*p* = 0.272) (**Figure 3B**). Furthermore, the postoperative difference in smiling distance between the operated and healthy sides did not significantly differ from preoperative values (Po_3M: *p* = 0.161; Po_6M: *p* = 0.756). In contrast, the postoperative difference in smiling commissure angle between the operated and healthy sides was significantly greater at 1 month compared to preoperative values (*p* = 0.011), indicating asymmetry of the angulus oris. Symmetry was restored at 3 mouths (*p* = 0.115) and 6 months (*p* = 0.368) postoperatively (**Figure 3C**), supporting the temporary nature of facial nerve injury and its subsequent recovery.

The average preoperative MIO was 44.00 ± 1.05 mm. All patients began continuous mouth-opening training using mouth openers at 2 weeks postoperatively. At 1 month postoperatively, the average MIO decreased to 27.11 ± 2.05 mm, indicating moderate trismus, which was significantly smaller than the preoperative value (*p* = 0.000). Subsequently, the MIO increased to 37.33 ± 2.84 mm at 3 months (*p* = 0.055), 40.33 ± 1.36 mm at 6 months (*p* = 0.056), and 43.11 ± 1.02 mm at 12 months postoperatively (*p* = 0.452), showing no statistically significant difference compared with preoperative measurements (**Figure 3D**).

These data suggested that following oral cancer defect reconstruction with t-FAMMIF, the facial nerve dysfunction and trismus were temporary and reversible, with no long-term adverse effects.

### 3.2 Construction and histological evaluation of the ipsilateral t-FAMMIF and SMIF model in beagle dogs

#### 3.2.1 Construction of the beagle dog model

**Figure 4:**
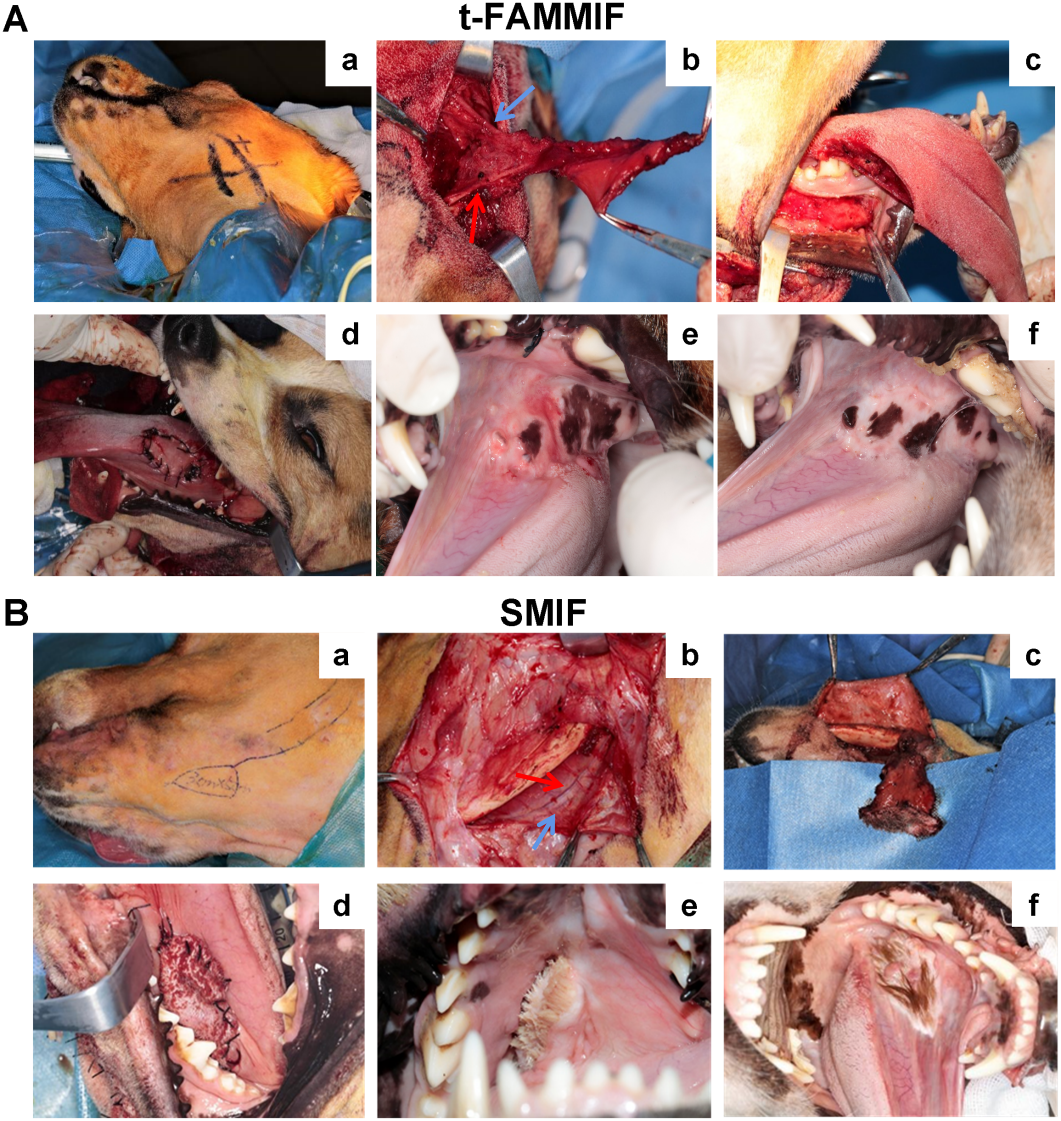
Surgical procedure of the t-FAMMIF and SMIF beagle dog model. **(A)** Construction of the t-FAMMIF beagle dog model: **(a)** Mark reference lines before surgery to indicate the surface projection of the FA and mandibular margin; **(b)** Display the facial artery (red) and facial vein (blue) respectively; **(c)** Defect site; d: The t-FAMMIF for repairing the tongue defect; **(e)** 4 weeks post-operation; f: 12 weeks post-operation. **(B)** Construction of the SMIF beagle dog model: **(a)** Mark the flap design; **(b)** Identify the submental artery (red) and submental vein (blue); **(c)** SMIF preparation; **(d)** SMIF for repairing the tongue defect. **(e)** 4 weeks post-operation; f: 12 weeks post-operation. t-FAMMIF, tunnelized-facial artery myomucosal island flaps; SMIF, submental skin island flap.

To investigate the differences between mucosal and cutaneous flaps, the SMIF was selected as a representative cutaneous flap, serving as a control group for t-FAMMIF. Both are pedicled flaps of comparable dimensions, ensuring high viability and facilitating a standardized comparison. Three weeks postoperatively, there was no flap loss or necrosis in either the t-FAMMIF or SMIF group. After the procedure, the dogs could eat independently and showed no impairment in daily movement. Thus, the animal model was successfully established. The surgical process is shown in **Figure 4**.

#### 3.2.2 Histological evaluation

**Figure 5:**
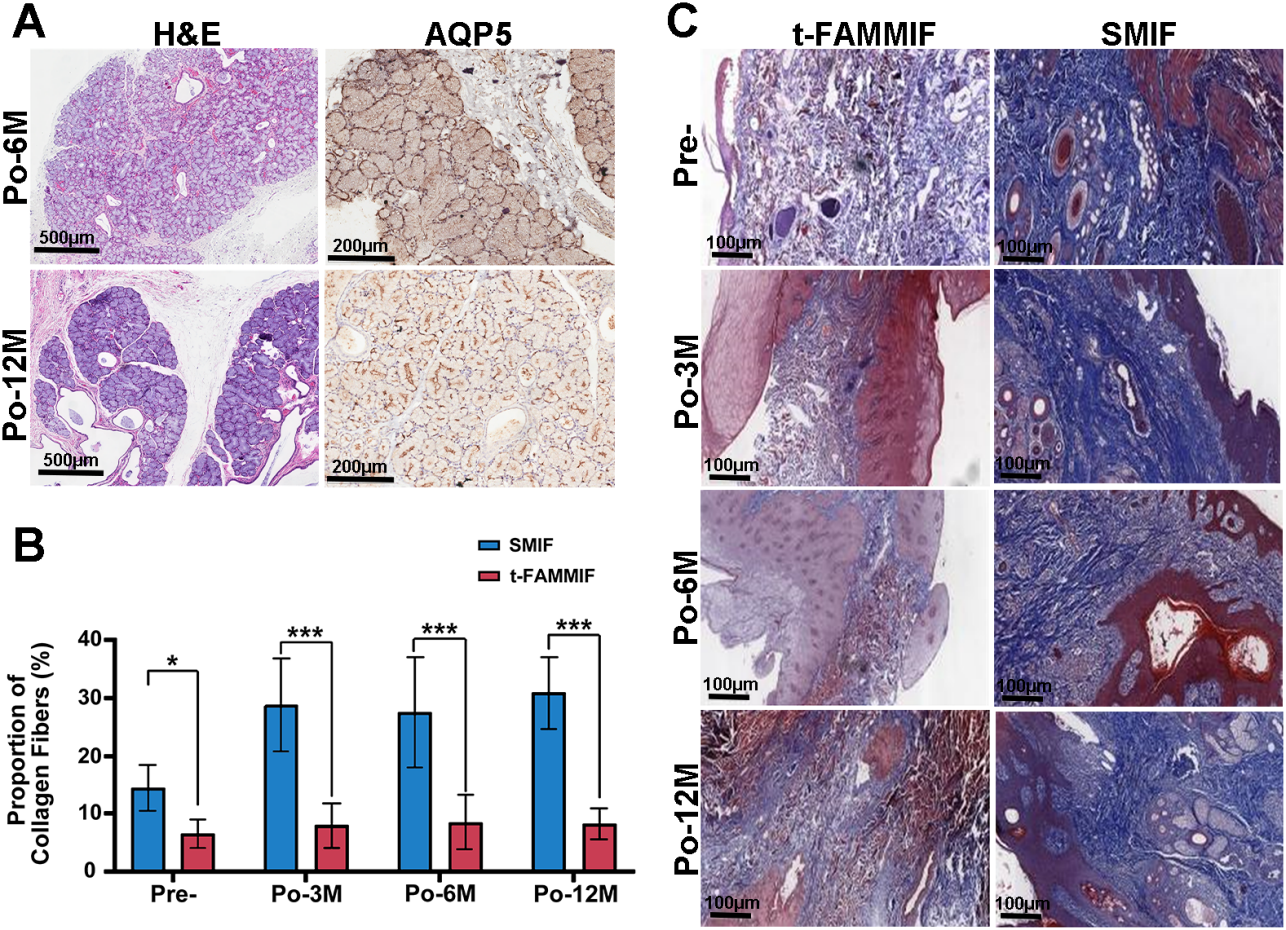
Comparison between the t-FAMMIF and SMIF. **(A)** H&E staining of minor salivary gland and IHC staining of AQP5 in the t-FAMMIF group (20×). **(B)** Comparison of Masson staining results between the t-FAMMIF and SMIF group. **(C)** Masson staining of the t-FAMMIF and SMIF group (20×). Pre-, preoperatively. Po-3M to Po-12M, 3 to 12 months post-operatively. AQP5, aquaporin-5. t-FAMMIF, tunnelized-facial artery myomucosal island flaps. SMIF, submental skin island flap. (***, *p* ≤ 0.001. *, *p* ≤ 0.05).

The histological results revealed that the minor salivary glands had normal morphology, with well-defined glandular structure, distinct branching ducts, and intact capsules (**Figure 5A**). Aquaporin-5 (AQP5) expression was observed on the apical, lateral, and basal membranes of the acinar cells, indicating the preserved tissue architecture and glandular function (**Figure 5A**). This evaluation was conducted as a descriptive analysis to characterize the glandular features within the mucosal flaps.

Masson staining demonstrated a marked accumulation of blue-stained collagen fibers in the dermis of the SMIF group whereas the t-FAMMIF group showed a significantly lower density of collagen fibers in the lamina propria. Although intergroup differences were preoperatively evident (*p* = 0.030), they became more pronounced following the postoperative scar formation (*p* < 0.001; **Figures 5B, C**). Consequently, compared to the cutaneous flap, the mucosal flap exhibited less scar contracture and a softer texture.

## 4 Discussion

This study demonstrated favorable postoperative functional recovery with manageable complications in patients treated with t-FAMMIF. While previous studies have reported clinical outcomes of FAMM flaps (16–18), this study offered two key advancements. First, facial nerve function in t-FAMMIF patients was evaluated using multiple modalities, incorporating both objective and quantitative assessments. Second, histological analysis in an animal model was conducted to elucidate the mechanisms underlying the notable sensory recovery observed in the t-FAMMIF group. To our knowledge, this is the first study to directly link tissue-level characteristics with functional outcomes.

For patients with a malignant tumor, oncological safety remains the primary concern. Although cutaneous flaps have been widely applied in the reconstruction of defects following surgery for oral cancer, the long-term exposure of cutaneous flaps to the oral environment may result in the loss of the stratum corneum and skin appendages, chronic inflammation (3), epithelial dysplasia (4), and even malignant transformation (5, 6). Kadakia et al. reported a higher complication rate for cutaneous flap-based repair of mucosal defects than that for skin defects, which suggests that cutaneous flaps are more suitable for repairing skin defects rather than mucosal defects (19). The skin and mucosa adapt to distinct environments, and this makes mismatched repairs suboptimal. In 1957, Harold Gilies proposed a key principle of plastic surgery: “losses must be replaced in kind (20)”. Therefore, skin should be used for skin defects and mucosa for mucosal defects. The restoration of the preoperative state as much as possible through homologous restoration is the ultimate goal of reconstructive surgery.

For patients with oral cancer, tumor treatment is the primary priority, followed by efforts to enhance their quality of life. The University of Washington Quality of Life (UW-QoL) questionnaire identified “Appearance,” “Recreation,” and “Chewing” as key factors that influenced the postoperative quality of life in these patients (21). Reconstructive surgery played a significant role in improving postoperative well-being (22). This highlights the importance of considering both aesthetic and functional aspects in reconstructive procedures. Nonetheless, the role of aesthetics in the oral cavity is equally important and should not be overlooked. Compared to cutaneous flaps, t-FAMMIF minimizes the risk of inflammation and malignant transformation, is hairless, and resembles the normal mucosa, and therefore confers favorable aesthetic outcomes. In a study by Joseph et al., FAMM demonstrated superior aesthetics and reduced donor-site morbidity (23). In some of our cases, surgical scars were nearly imperceptible, which constitutes the ideal reconstruction outcome.

The soft tissues of the tongue and the floor of the mouth are functionally essential for mobility, speech, mastication, and deglutition. Reconstructing these areas thus presents unique challenges for transplanted tissue. Compared to cutaneous flaps, the t-FAMMIF is thinner, softer, and easier to shape, resulting in fewer scars. This may contribute to superior mobility, enhanced recovery of deglutition, speech, and overall oral function. Especially in the reconstruction of small and delicate structures, such as the tip of the tongue, the outcome depends heavily on the flap’s thinness, pliability and the preservation of tongue mobility (9). To directly compare the mucosal and cutaneous flaps, we established animal models of t-FAMMIF and SMIF, and the results confirmed that mucosal flap exhibited greater pliability and facilitated improved mobility.

Previous studies report tactile sensation recovery rates of 96.1% to 100% for t-FAMMIF, compared to only 20% to 70% for cutaneous flaps (24–26). Consistent with these findings, our t-FAMMIF group achieved 100% recovery of both tactile sensation and two-point discrimination at 12 months, outperforming the cutaneous flap group. Previous reports speculated that this enhanced sensory recovery stems from reduced fibrosis and contraction in the t-FAMMIF, which facilitates nerve sprouting from adjacent tissues (25). Our histological data directly substantiate this mechanism. We propose that minimized scarring generates a highly permissive microenvironment for nerve growth, explaining the rapid and superior tactile recovery observed in our clinical cohort. Furthermore, minor salivary glands were well preserved within the mucosal flap, enabling continuous salivary secretion that prevents surface desiccation and may further contribute to a favorable healing environment.

The clinical application of t-FAMMIF should be guided by the following stringent criteria: (a) an appropriate defect size, and (b) no evidence of lymph node metastasis. In our practice, we found that the t-FAMMIF was suitable for defects up to 7 cm × 5 cm, which approximately corresponds to the postoperative defect size in T2–T3 oral cancer. Although the contralateral t-FAMMIF further minimizes oncological risk (27), the ipsilateral t-FAMMIF remains a practical and safe alternative when metastasis is clinically absent. Patients with occult nodal metastases (pN+) identified postoperatively received adjuvant radiotherapy. As prior studies reported, preservation of the facial vessels did not compromise regional control (28, 29). Our results showed no signal of increased oncologic risk associated with t-FAMMIF, with comparable 3-year progression-free survival rates between the t-FAMMIF group and cutaneous flap group (95% *vs* 95%, *p* = 0.92). However, one patient in our t-FAMMIF group developed ipsilateral cervical lymph node metastasis. Therefore, given the small sample size, these findings should be interpreted as preliminary evidence of oncologic safety, rather than definitive proof of equivalence. Furthermore, this approach is particularly advantageous for patients requiring concurrent cervical lymphadenectomy, as it avoids redundant scarring. Key contraindications for t-FAMMIF include cases that involve damage to or absence of the facial vessels, a history of radiation therapy, and oncological risk.

Regarding the management of complications, although t-FAMMIF may cause facial nerve dysfunction and trismus, these outcomes are preventable or reversible. Our findings are largely consistent with those of previous studies (16, 17). The transient facial nerve dysfunction is likely attributable to neuropraxia from temporary traction on the marginal mandibular branch during tunnelization and flap inset, rather than structural nerve injury. Intraoperatively, protection of the facial nerve should be prioritized. To ensure its visibility throughout the procedure and thereby facilitate its protection, the marginal mandibular branch of the facial nerve should be identified and exposed prior to the exposure of the facial artery and vein. Postoperatively, nerve nourishment and rehabilitation therapy should be initiated. Timely and effective mouth-opening exercises are crucial, and it is recommended that sustained training using a mouth opener should commence 2 weeks after surgery. Moreover, it is essential to emphasize that poor patient compliance can result in permanent trismus.

This study demonstrates favorable outcomes of t-FAMMIF in oral cavity reconstruction and suggests potential advantages over cutaneous flaps in sensory recovery. Nevertheless, several limitations must be acknowledged. First, as a single-center study with a modest sample size, its statistical power was limited, particularly for oncologic outcomes with few events. Second, the matching model did not include other potential confounders due to sample size constraints, which may have introduced residual confounding. Third, assessment of speech and tongue mobility relied on qualitative observations without validated objective measurements. Fourth, the animal experiment, though providing valuable mechanistic insights, should be considered exploratory due to its limited scale. Therefore, large, prospective randomized controlled trials with comprehensive functional assessments are necessary to further validate these findings.

In conclusion, the t-FAMMIF offers an effective option for the reconstruction of moderate-to-small mucosal defects following tumor resection in the head and neck region. Compared to cutaneous flaps, the t-FAMMIF provides several advantages, including improved aesthetic outcomes, superior sensory recovery, and the preservation of salivary gland function.

## Data Availability

The raw data supporting the conclusions of this article will be made available by the authors, without undue reservation.
