# Supplementary material for: Clinical outcomes of the tunnelized-facial artery myomucosal island flaps in the oral cavity and comparison with cutaneous flaps in an animal model
Source: Front Oncol. 2026 Apr 7;16:1760967. doi: 10.3389/fonc.2026.1760967 (PMC13095581; doi:10.3389/fonc.2026.1760967)
Supplement: Supplementary file 1 [file DataSheet1.docx]

Supplementary Material

# Supplementary Data

Supplementary Material should be uploaded separately on submission. Please include any supplementary data, figures and/or tables.

Supplementary material is not typeset so please ensure that all information is clearly presented, the appropriate caption is included in the file and not in the manuscript, and that the style conforms to the rest of the article.

# Supplementary Figures and Tables

## Supplementary Figures

**
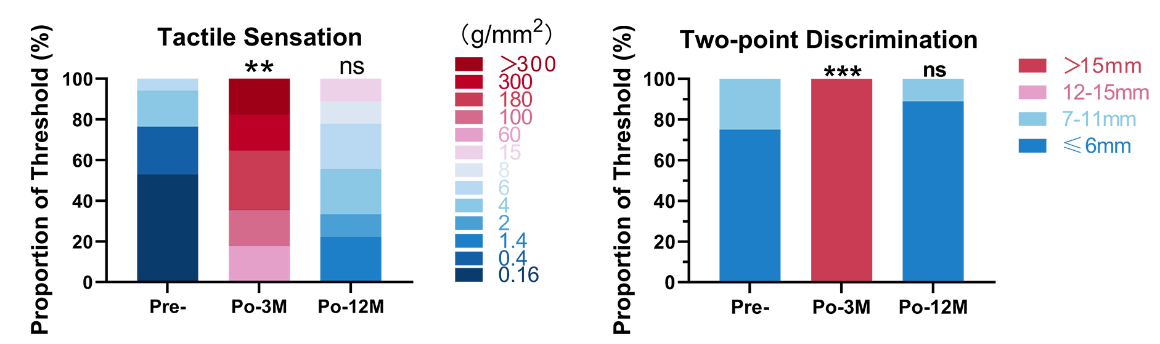
**

**Supplementary Figure 1.** Comparison results of sensory function assessment before and after surgery in t-FAMMIF group. (***, *p* ≤ 0.001. **, *p* ≤ 0.01. ns, *p* > 0.05)

## 2.2 Supplementary Tables

**Supplementary Table 1.** **Comparison results of sensory function assessment before and after surgery in t-FAMMIF group.**

|  | **Pre-** | **Po-3M** | **Po-3M  *p* value** | **Po-12M** | **Po-12M  *p* value** |
| --- | --- | --- | --- | --- | --- |
| **Tactile Sensation (g/mm2)** | 1.24 (1.92) | 240.00 (189.34) | 0.010** | 5.31 (4.29) | 1.000 |
| **Two-point Discrimination (mm)** | 4.88 (1.93) | 15.00  (0.00) | 0.000*** | 4.78 (2.05) | 0.297 |

Mean (standard deviation). Pre-, preoperatively. Po-3M to Po-12M, 3 to 12 months post-operatively. (***, *p* ≤ 0.001. **, *p* ≤ 0.01)
